# Breastfeeding counseling based on formative research at primary healthcare Services in Mexico

**DOI:** 10.1186/s12939-021-01491-6

**Published:** 2021-07-27

**Authors:** Diana Bueno-Gutiérrez, Edgar Uriel Romero Castillo, Angélica Emili Hernández Mondragón

**Affiliations:** UABC, Calzada universidad 14418, parque industrial internacional, CP 22390 Tijuana, BC Mexico

**Keywords:** Breastfeeding, Socio-ecological, Primary healthcare level, Mexico

## Abstract

**Background:**

Breastfeeding rates in Mexico are far from World Health Organization (WHO) recommendations with 28.8% of Exclusive Breastfeeding (EBF) under 6 months of age, according to the 2018 National Health and Nutrition Survey. Formative research has shown that culturally appropriate counseling is an effective breastfeeding intervention. The objective of the current study was to evaluate the effect of interpersonal counseling on EBF in a primary healthcare center in Tijuana, México.

**Methods:**

This was a randomized controlled trial pilot with a sample of mothers with infants under 4 months of age from a primary care center. Participants were randomized into two groups: 1) Control group, received counseling on immunizations and standard infant feeding information, and 2) Intervention group, receiving breastfeeding counseling using a socio-ecological framework. Changes in breastfeeding attitudes, self-efficacy and EBF were evaluated at 2 months post-intervention.

**Results:**

A total of 80 mothers completed the 2 month follow up assessment (40 in each group). The mean age at baseline was 26.4 years for mothers and 1.4 months for infants. There was a 30% increase in EBF at 2 months follow up in the intervention group and 15% decrease in the control group post-intervention. We observed a significant improvement in breastfeeding attitudes (*P* = 0.0001), self-efficacy (*P* = 0.046) and EBF (P = 0.0001) in the intervention group. Reported obstacles were discomfort of breastfeeding in public (23%), infant dissatisfaction (23%), pain (19%), insufficient milk supply (15%) and returning to work (8%).

**Conclusions:**

Breastfeeding counseling based on previous formative research improved breastfeeding attitudes, self-efficacy and practices in this population. These findings suggest that the promotion of breastfeeding utilizing a socio-ecological framework may improve breastfeeding rates by addressing the needs of women within their varying sociocultural contexts.

**Trial registration:**

ACTRN: ACTRN12621000915853. Retrospectively registered.

**Supplementary Information:**

The online version contains supplementary material available at 10.1186/s12939-021-01491-6.

**Table:** 

This article is a part of the Interventions and policy approaches to promote equity in breastfeeding collection, guest-edited by Rafael Pérez- Escamilla, PhD and Mireya Vilar-Compte, PhD	

## Background

Breastfeeding is the single most cost-effective intervention to prevent child mortality [1]. The benefits of breastfeeding are beyond survival and include better child development and improved maternal health [2–5]. However, the overall rate of exclusive breastfeeding (EBF) for infants under 6 months of age is only 40%. In the Americas, less than 6% of countries have an EBF rate above 60% [6]. Even though Mexico increased EBF from 14.4% in 2012 to 28.6% in 2018, rates remain suboptimal. The use of infant formula is particularly prevalent in northern Mexico, with 51.7% of mixed feeding (both infant formula and breastfeeding) being reported, higher than the national mean of 25.6% [7, 8].

Interpersonal counseling (IC) has proven to be an efficient intervention to change breastfeeding rates [9, 10]. In a systematic review conducted in 2018 [10], in which 63 interventions were analyzed, IC was associated with a decreased risk of early termination of EBF at 4–6 weeks (RR =0.79, 95% CI 0.72, 0.87) and at 6 months (RR = 0.84, 95% CI, 0.78, 0.91). More than 75% of the studies were done in high income countries, highlighting the importance of conducting interventions in low- and middle-income countries to evaluate the effects of counseling in different contexts.

To scale up breastfeeding programs more efficiently in health systems, the gear model delineates the integration of key aspects, such as promotion, training, research and program design [11]. An evaluation based on this model was conducted in Mexico in 2016 [12], resulting in the following recommendations: 1) community needs assessments, formative research, and pilot studies that take into consideration the key actors working in different sectors, 2) the development of primary care breastfeeding services in the health system, 3) training of health professionals that not only focuses on theoretical fundamentals, but also on communication and problem solving practical skills, 4) integration of breastfeeding interventions into existing health programs such as immunization services, and 5) intervention monitoring and outcome evaluation.

This study is the continuation of a project that originated from formative research to evaluate breastfeeding obstacles in Tijuana’s low-income communities [13]. A socioecological framework was used to generate 10 main obstacles, which were organized as follows: 1) individual factors, such as pain or perception of insufficient milk (PIM); 2) group factors, such as a lack of support from family members, health services and work environment; and 3) social factors, such as discomfort from breastfeeding in public.

**Fig. 1:**
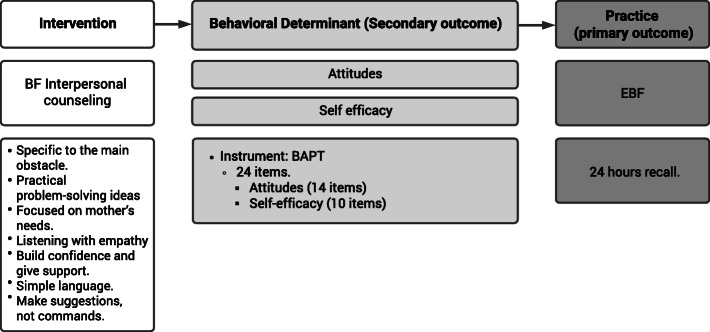
Behavioral change model used for intervention

We adopted the theory of planned behavior (TPB) as a model for behavioral change (Fig. 1) to design educational material with the aim of overcoming these 10 breastfeeding obstacles (Table 1). According to TPB, the intention to perform a behavior is influenced by an individual’s attitudes, perceived behavioral control (self-efficacy), and subjective normative beliefs [14]. The 10 health messages were tested in a sample of mothers with children under 2 years of age. Our results indicated that after being exposed to a brief presentation (1–2 min per message), there was a significant change toward more positive breastfeeding attitudes but no effect on breastfeeding intentions and self-efficacy [15]. Improvements were made to increase self-efficacy, such as adding more ideas to solve practical problems, and integrating IC focusing on the main obstacle each mother had concerning breastfeeding. More information about educational material design and testing is presented in Box 1 and Additional file 1.

**Table 1:** Breastfeeding messages based on formative research

SEF	Breastfeeding obstacles	Messages
Individual Factors	Pain	If you ever feel pain, go over your posture and seek the help of a lactation consultant. Pain must be gone over the first days
	Perception of insufficient milk	Breast milk is the ideal food for babies’ growth and development and it’s the only source of food they need during the first six months of life
	Infant dissatisfaction	When babies cry, it’s not because they don’t like breast milk. Babies’ tears are a way to communicate their needs. It’s possible that they are uncomfortable or maybe he just wants a hug, for some air to be cleared up or his diaper changed
	Mothers discomfort	Breast milk is the only food that is always ready for the baby. Breastfeeding women save time, money and energy. They don’t have to spend time preparing formula and a healthier baby will visit the pediatrician less frequently
	Time constraints	
	Esthetics	Some women are worried about breastfeeding causing their breasts to sag. But this is not true. Breasts changes through pregnancy regardless of how the baby is fed.
Group Factors	Family support	Breastfeeding can be an opportunity to rest. Spouse, partners and family members can facilitate this by encouraging the mother to lie down in a peaceful place.
	Support at work	There are several ways to continue breastfeeding once you get back to work. Inform yourself about the laws that protect your rights. Learn to express and store your breast milk so that your baby continues receiving its benefits
Social Factors	Breastfeeding in public	Babies have the right to be breastfed whenever and wherever they’re hungry
	Formula culture	To regain our ancestors’ knowledge on breastfeeding, is to regain our children’s health

*SEF* Socio-ecological framework

The objective of this pilot study was to evaluate the effect of interpersonal counseling on exclusive breastfeeding rates in infants under 6 months of age at a primary healthcare center in Tijuana, Mexico.

**Table:** 

**Box 1** Educational material design, testing and application	
- We used qualitative methods to design messages based on a socio-ecological framework - Content development: Messages were designed by a panel of public health professionals and lay women; - Message refining and Cognitive testing: Qualitative methods were used to identify message preferences for mothers. There were 5 brochures for obstacles with more practical information - Pain: How to get a good latch, when do you need to ask for professional support - PIM: How do you know you are producing enough milk for your baby - Infant dissatisfaction: Understand 3 normal infant behaviors: Crying and hunger-satiety cues, sleep, breastfeeding on demand (No schedule setting) - Family support: How Dad/Grandparents can support a breastfeeding mother - Work: Rights and regulations, milk expression	
For the other 5 obstacles with a more psychological-social content we used posters with a main message and a picture with a “real breastfeeding mom” from Tijuana. These images were taken with permission from a local campaign and were pre-tested.	
For more information see Appendix 1/Reference 13	
**General description of the Interpersonal counseling session** The medical intern tells the nurse about the main obstacle of the participant mother. The nurse selects the corresponding educational material (brochure/ poster). He begins by asking open-ended questions to understand the mothers´ context and then focuses on listening, empathizing and giving practical solutions to her obstacle. At the same time, the nurse works at creating a friendly and safe environment. He must pay attention to the baby’s´ turn to get vaccinated, because if the mother is worried about missing their turn, she will be tense and not attentive to the counseling.	

## Methods

### Design and setting

This randomized [1:1] controlled pilot trial was conducted at a primary health care center in Tijuana, México, from October 2018 to March 2019. The study was approved by the Ethics Committee from the Facultad de Medicina y Psicología (FMyP) at Universidad Autonoma de Baja California (UABC) and by the Sanitary Jurisdiction No. 2.

The health center is part of Baja California’s Health System, which generally offer services to the uninsured population. To carry out this pilot, we selected a center with a good inflow of people located in a strategic area in Tijuana. Mothers were approached when they brought their infants to be immunized. This allowed us to integrate the intervention to a pre-existing health service increasing the probability to become a sustainable program.

**Fig. 2:**
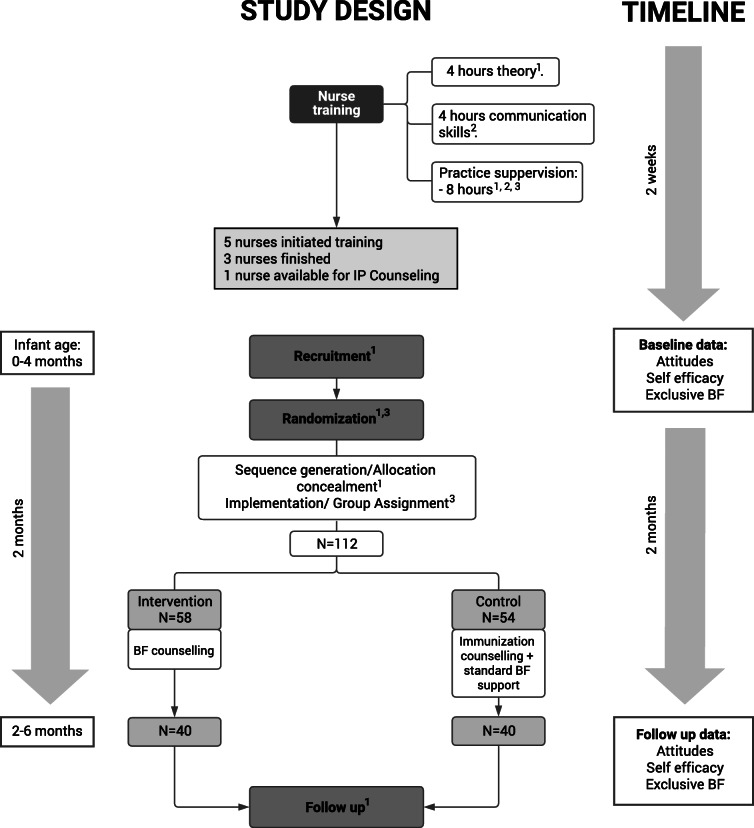
Study design flowchart and timeline

The study design and timeline is shown in Fig. 2.

### Participants

Mother-infant dyads that met the following inclusion criteria were selected: 1) requesting immunization services at the health center, 2) mothers ≥18 years of age, and 3) infants ≤4 months. This last criterion was established, considering that the outcome would be measured 2 months after the intervention ensuring proper assessment of EBF before 6 months old. The exclusion criteria were: 1) infants with low birth weight (< 2500 g), 2) infants with < 38 gestational weeks, 3) twins, and 4) maternal or infant contraindications to breastfeeding.

### Intervention

The intervention consisted of providing IC, which focused on solving the main breastfeeding obstacle identified by the mother. The information provided included brochures and posters (Box 1, Additional file 1), explained by a nurse to the mother before the infant was immunized. The control group received immunization counseling and standard breastfeeding support.

Five nurses were trained for IC (Fig. 2). First, a 4-h session was held, in which 2 medical interns (EURC, AEHM) provided general background on breastfeeding, as well as information about the 10 main obstacles to breastfeeding identified from previous formative research [13]. A second 4-h session was held guided by a community promoter focused on the WHO breastfeeding counseling training course [16]. The focus of this session was to develop communication skills for “Listening and Learning” (open questions, empathize, avoid judging words) and “Building Confidence and Give Support” (accept what a mother thinks and feels, recognize and praise what she is doing right; give small bits of relevant information; use simple language; make suggestions, not commands).

To evaluate nurses´ counseling performance, supervised practice sessions were conducted with mothers from the target population, until consistency between trainees was determined. Due to the workload of the health center, only 3 nurses completed the training, and only 1 was able to participate in the intervention.

### Study process: recruitment, randomization, follow-up

Mothers with infants requesting immunization services were approached in the waiting room of the health center and given the necessary information about the study. If mothers showed interest in participating, informed consent was presented to be read and signed. Subsequently, a questionnaire was applied to obtain sociodemographic data, as well as instruments to assess attitudes, self-efficacy and breastfeeding practices. The following step was to identify their main obstacle to breastfeeding from a list with the 10 obstacles established in previous formative research [13]. Two additional options were available: adding another obstacle or expressing not having a breastfeeding problem.

A computer generated sequence of random numbers and allocation concealment was conducted by the principal investigator (DBG). Sealed envelopes for each corresponding number were delivered to the medical interns at the health care center. Each time a new dyad was recruited, the medical interns opened the corresponding sealed envelope to assign the dyad to their group. Participants assigned to the intervention group received one session of breastfeeding IC by the trained nurse lasting between 5 and 10 min. Participants assigned to the control group received counseling on immunizations. Both groups were scheduled for their next vaccination dose for 2 months afterward. Follow-up data were obtained by the medical interns at the subsequent visit service. If participants did not attend the 2 month follow-up, they were contacted by telephone to assess attitudes, self-efficacy, and breastfeeding practices.

### Outcome assessment

#### Breastfeeding practices

The primary outcome was EBF 2 months after the intervention. The definition of EBF utilized was: “only breast milk or prescribed medications/vitamin supplements, without the use of water, juices, formula or solid food” [17]. The evaluation was made based on a 24-h recall. Other measured outcomes were any other type of breastfeeding and the main 3 obstacles to breastfeeding.

#### Breastfeeding attitudes and self-efficacy

To measure attitudes and self-efficacy toward breastfeeding, two subscales of the “Breastfeeding Attrition Prediction Tool (BAPT)” [18–20] were used: negative attitudes toward breastfeeding and lactation control (self-efficacy). The BAPT was developed using the TPB to predict breastfeeding attrition. It uses a 5-point Likert-type scale that ranges from strongly agree to strongly disagree. The 14 items used for negative attitudes are listed in Table 4. For the control subscale, 10 items that reflect the woman’s ease or difficulty to breastfeed were used. Items included were: I have the skills to breastfeed, I know how to breastfeed, I am determined to breastfeed, I am physically able to breastfeed, I am confident I can breastfeed, I have total control to breastfeed, I am ready to breastfeed, I have enough milk, and breastfeeding is easy.

The construct and predictive validity of the scale and subscales have been previously determined in their original version in English [18, 19] and a version translated into Spanish [20]. Internal consistency has been estimated with a Cronbach’s alpha of 0.784 for the negative attitude subscale and 0.864 for the control subscale [19]. The test-retest reliability was evaluated by this research group in a sample of women with similar characteristics to the study population. We obtained an intraclass correlation (ICC) of 0.65 for negative attitudes and 0.78 for control subscale [15].

### Co- variables

Other variables were organized as follows: 1) sociodemographic (occupation, marital status, level of education, place of birth, length of time living in Tijuana), 2) mothers´ information (number of children, type of delivery of the last child, other information received about breastfeeding prior to recruitment); and 3) infant data (date of birth, sex, birth weight, and weeks of gestation at birth).

### Data analysis

Based on the population attending immunization services at the health center, it was determined to use a sample of 50 mothers for the intervention group and 50 for the control group. This sample size provided 80% power to appreciate a change of 20% in EBF prevalence between groups.

We used SPSS v21 for data analysis. Descriptive statistics were used to measure central tendency and dispersion for continuous variables and frequencies for categorical variables. To determine negative attitudes and control scores, we assigned for each item 1 point for “strongly disagree” and 5 points to “strongly agree”. Higher scores indicate a greater sense of control and higher negative attitudes. We used 14 items from the negative attitudes subscale and 10 items from the control subscale.

To evaluate the effectiveness of the intervention, we used 1) chi-square for EBF and Student’s t-test for attitude and self-efficacy scores and, 2) regression models, linear for continuous variables (attitudes and self-efficacy) and logistic for EBF.

## Results

### Descriptive data

We reached 80 participants at the end of the 2 month follow-up period from 112 at baseline (71% retention). There were no significant differences when comparing characteristics of the remaining participants vs those who were lost at follow-up, except for marital status, with a higher proportion of single women in the former (18% vs. 6%, *p* = 0.02). The mean age at baseline was 26.4 years for mothers and 1.4 months for infants. The proportion of infants ≤1 month-old was 49%, 1–3 months 27%, and 3–4 months 24%. The majority of mothers in both groups were homemakers (73%), living with partners (63%), finished middle school (42%) and or high school (32%), and had a vaginal birth (60%) (Table 2).

**Table 2:** Maternal and infant baseline characteristics of the study groups

	Intervention *n* = 40		Control *N* = 40		*P*
	Mean	SD	Mean	SD	
Maternal age (y)	27.1	7.3	25.6	5.9	0.31
Infant age (m)	1.65	1.5	1.23	1.5	0.21
Number of children	2	1.2	1.93	1.1	0.77
Birthweight (kg)	3.3	0.5	3.3	0.4	1.0
Gestational age at delivery (w)	39.3	1.3	39.3	1.1	1.0
	Intervention		Control		*P*
	%	n	%	n	
C-section	35	14	45	18	0.49
Primiparous	48	19	48	19	1.00
Female infant	53	21	55	22	0.82
Previous BF information	80	32	58	23	0.05
Marital Status					
Living with partner	60	24	65	26	0.69
Married	18	7	20	8	
Single	22	9	15	6	
Occupation					
Homemaker	80	32	68	27	0.46
Employed	20	8	22	9	
Student	0	0	10	4	
Education					
Elementary	5	2	10	4	
Middle school	45	18	40	16	0.63
High school	35	14	28	11	
University graduate	15	6	22	9	
Main obstacles					
Infant dissatisfaction	23	9	23	9	
BF in public	23	9	23	9	
Pain	18	7	20	8	
PIM	15	6	15	6	
Work	8	3	8	3	
None	8	3	8	3	0.88

Data are mean ± SD values or percentage (n), for subjects with known information, as indicated

*BF* Breastfeeding, *PIM* Perception of Insufficient Milk

The 112 participants were randomly assigned to intervention (*n* = 58) and control (*n* = 54) groups. Eighteen participants in the intervention and 14 participants in the control group could not be reached at 2 months follow up. The final number of participants in each group was 40 (Fig. 2). Baseline demographic characteristics were similar between them, except for the variable expressing the breastfeeding information received previous to this study. There was a difference reaching statistical significance (*P* = 0.05) between groups, with 80% of mothers reporting having received this information in the intervention group versus 58% in the control group. This difference was considered and added to the regression models for adjustment. No significant differences were detected in EBF, attitudes, or self-efficacy between groups at baseline (Table 3).

**Table 3:** Differences in Breastfeeding Attitudes, Self-efficacy and Exclusive Breastfeeding (EBF) between groups

Outcome	Baseline			Follow-up		
	**Intervention** **Mean ± SD**	**Control** **Mean ± SD**	***P***	**Intervention** **Mean ± SD**	**Control** **Mean ± SD**	***P***
**Negative Attitudes** ^a^	38.9 ± 6.5	40.3 ± 7.2	0.39	29.8 ± 8.4	40.3 ± 7.3	0.0001
**Self-efficacy** ^a^	38.4 ± 5.5	39.5 ± 5.0	0.36	41.7 ± 6.0	38.6 ± 5.2	0.018
	**% (n)**	**% (n)**	***P***	**% (n)**	**% (n)**	***P***
**EBF** ^b^	40 (16)	37.5 (15)	0.82	70 (28)	22.5 (9)	0.0001

^a^ *t* test

^b^ chi square test

The main obstacles reported by participants were infant dissatisfaction (23%), breastfeeding in public (23%), pain (19%), PIM (15%), and return to work (8%). When asked about a secondary obstacle, the culture surrounding breastfeeding appeared in fifth place (6%), in a similar proportion as return to work. The majority of mothers (92%) expressed at least one obstacle, 77% expressed two obstacles, and 44% expressed three obstacles.

### Main outcome: exclusive breastfeeding

At the 2-month follow-up evaluation, EBF was significantly higher in the intervention group than in the control group (70% vs. 22.5%, *P* = 0.0001) (Table 3). Logistic regression showed a significant increase in EBF with intervention (B 2.4, OR 10.7 95% IC 3.4, 33.0, *P* = 0.0001) (Table 4). The main change was observed in the infant age group from 0 to 2 months, with a pre/post intervention increase in EBF of 33% (42 to 75%), 20% in the 2–4 month group (47 to 67%), and 17% in the 4–6 month group (33–50%).

**Table 4:** Association between breastfeeding counseling intervention and breastfeeding attitudes, self-efficacy and exclusive breastfeeding (EBF)

**Outcomes**	**β (95% CI)**	***P***
**Negative Attitudes** ^a^	−11 (−14.6, −7.4)	0.0001
**Self-efficacy** ^a^	2.6 (0.04, 5.2)	0.046
	**OR (95% CI)**	***P***
**EBF** ^b^	10.7 (3.4, 33.0)	0.0001

^a^ Linear regression adjusted for breastfeeding information received previous to the study

^b^ Logistic regression adjusted for breastfeeding information received previous to the study

### Secondary outcomes: attitudes and self-efficacy

There were significant differences in changes in attitudes and self-efficacy between the intervention and control groups. Comparisons using t-tests showed that participants in the intervention group had decreased negative attitudes toward breastfeeding (*P* = 0.0001) and increased self-efficacy (*P* = 0.018) compared with the control group (Table 3). Linear regression models indicated a significant effect of the intervention on negative attitudes (P = 0.0001) and self-efficacy (*P* = 0.046) (Table 4).

There were changes regarding negative attitudes in every item of the subscale (Table 5). An improvement in negative attitudes in the intervention group is represented as an increase in the percentage of disagreement with breastfeeding negative attitudes. We reported this as the “final difference”, in which the difference from the control group to the intervention group was subtracted. For example, item 14 states that formula-fed babies are easier to satisfy. At baseline, 32.5% of mothers from the intervention group indicated that they strongly disagree or disagree (SD/D) with the statement. After the intervention, 70% of mothers from the same group expressed SD/D, indicating an increase of 37.5%. In the control group, 27.5% of mothers reported SD/D at baseline and 30% at the end, resulting in a pre/post difference of 2.5%. The final difference for item 14 is calculated by subtracting the 2.5% of the control group from the 37.5% of the intervention group, resulting in 35%. The negative attitudes that showed a higher level of disagreement at follow-up in the intervention group compared to control were: item 4, feeding with formula is easy; item 14, babies fed with formula are easier to satisfy; item 13, feeding with formula helps the father to be closer to his baby; and item 3, breastfeeding makes the breasts sag.

**Table 5:** Changes in Mothers´ Negative Attitudes

Item Negative Atttitude		Intervention %SD/D		Control %SD/D		Pre/Post Differences		Final Difference
		Pre	Post	Pre	Post	Inter- vention	Control	
1	BF ties you down	70	97.5	77.5	80	27.5	2.5	25
2	It’s hard to BF in public	42.5	72.5	40	45	30	5	25
3	BF makes your breast sag	47.5	75	62.5	60	27.5	−2.5	30
4	FF is easy	30	70	35	27.5	40	−7.5	47.5
5	FF mothers get more rest	47.5	75	42.5	57.5	27.5	15	12.5
6	BF is time consuming	82.5	85	70	67.5	2.5	−2.5	5
7	BF makes going to work hard	35	65	10	12.5	30	2.5	27.5
8	BF causes pain	52.5	80	35	40	27.5	5	22.5
9	No one can help if you BF	35	67.5	47.5	57.5	32.5	10	22.5
10	FF mothers get into shape	60	82.5	62.5	62.5	22.5	0	22.5
11	BF is messy	57.5	77.5	67.5	60	20	−7.5	27.5
12	Hard to know if enough milk	62.5	82.5	55	52.5	20	−2.5	22.5
13	FF lets dad closer to baby	50	75	50	37.5	25	−12.5	37.5
14	FF babies are easier to satisfy	32.5	70	27.5	30	37.5	2.5	35

*BF* Breastfeeding, *FF* Formula feeding

SD: Strongly Disagree, D: Disagree

### Obstacles

The sample size was inadequate for subanalysis of the associations between breastfeeding obstacles and EBF, attitudes and self-efficacy. Despite this limitation, we describe the changes observed in the 5 most frequent obstacles (Table 6). The most noticeable changes occurred in the case of pain, with a 29% pre/post increase in EBF (from 43 to 72%) in the intervention group and a decrease of 37% (from 50 to 13%) in the control group. Even though breastfeeding in public started with high rates of EBF in both groups (67%), it increased to 89% in the intervention group, and it decreased to 33% in the control group.

**Table 6:** Changes in EBF according to main obstacle

	EBF Intervention		EBF Control		Negative Attitudes	% Disagreement Post- Intervention^a^	
	Pre	Post	Pre	Post	Item	Intervention	Control
Pain	43	72	50	13	8	80	40
BF in public	67	89	67	33	2	72.5	45
Work	33	100	0	0	7	65	12.5
PIM	0	50	17	33	12	82.5	52.5

*BF* Breastfeeding

^a^ % Disagreement (strongly and just disagree) reported to the corresponding “Negative Attitude” item, after the intervention. For example for pain, 80% of mothers in the intervention group Disagreed with item 8: “BF causes pain” compared to 40% in the control group

### EBF according to obstacle

We observed consistency in the change of attitudes (according to specific BAPT items) and EBF in participants with the main obstacles: pain, breastfeeding in public, return to work, and PIM (there is no item related to baby dissatisfaction in BAPT). There was a higher increase in the percentage of disagreement of negative attitudes in the intervention group compared to the control group, coinciding with changes in EBF practices post intervention. Table 6 compares pre/post changes between the intervention and control groups in EBF and negative attitudes. For instance, in the case of pain, item No. 8 in the BAPT instrument used for this study (Table 5) states “BF causes pain.” At the end of the intervention, 80% of mothers receiving BF counseling reported disagreement (SD/D) with that statement, compared to 40% of mothers in the control group.

## Discussion

Following formative research that evaluated breastfeeding obstacles in low-income communities within the city of Tijuana in northern Mexico, the objective of this study was to evaluate the effect of IC on EBF in infants under 6 months of age. Results indicate an improvement in EBF, attitudes, and self-efficacy in participants from a primary health care center. There was a 30% increase in EBF in the intervention group, and a 22.5% decrease in the control group.

The primary obstacles reported for breastfeeding in this study align with the results of previous research from Mexico [13, 21, 22] and other countries [23, 24]. Even though participants had the option of adding obstacles that were not listed in the study questionnaire, all the participants who reported having some difficulty breastfeeding selected one of the 10 options presented. The 5 primary obstacles to breastfeeding in this study are similar to those that had already been identified in our previous investigation such as pain, PIM and breastfeeding in public. Infant dissatisfaction was noted more frequently in this pilot study, while aesthetic issues appeared less frequently than in our previous formative research.

Although the sample size was insufficient to perform the subanalysis among the obstacles related to breastfeeding, changes were most notorious with pain and discomfort from breastfeeding in public. Among the group of women who selected pain as their primary problem, there was an increase in EBF from 43 to 72% in the intervention group and a decrease from 50 to 13% in the control group. This highlights the importance of dealing with this frequent problem, which can be a compelling reason to stop breastfeeding, and one that particularly affects first-time mothers [25]. In this study, 24% of primiparous women expressed that pain was their primary problem, compared to 14% of multiparous women. Taking into account that in our study 48% of the women were first-time mothers, this could have influenced the observed effect on lactation.

Discomfort from breastfeeding in public was one of the main obstacles reported in 23% of the cases, but the baseline EBF levels were high (67%) among this subgroup. This might indicate that this is not an obstacle with a significant effect on breastfeeding cessation. However, at the 2-month follow-up, the intervention group presented 89% EBF and the control groups presented 33%, which shows the relevance of addressing the problem since if this is not considered, it could eventually lead to breastfeeding suspension.

This study underscores the importance of adopting horizontal models of education and support for breastfeeding. Models that take into account mothers’ needs may be more beneficial compared to practices that rely on reciting a list of benefits from breastfeeding or apply authoritarian styles in which health professionals tell mothers “what they should do” or even “scold them.” In this study, the counseling provided was based on previous formative research that assessed the main obstacles to breastfeeding in this population and the ways in which they would like to be supported. We used this information to create educational material and training methods for the nursing staff, aiming to solve the mother’s main breastfeeding problem with empathy and considering the mothers´ context.

Breastfeeding interventions have been recommended based on formative research. In Mexico, most of the studies on this topic have been conducted in the country’s central region. There is a classic intervention by Morrow et al. [22], in which exhaustive preliminary work was done to identify the obstacles to breastfeeding in a population in this region of the country. Subsequently, 3–6 sessions of home counseling took place and a significant increase in the duration and exclusivity of breastfeeding was obtained (*p* = 0.02). In a more recent study, Monterrosa et al. [26] reported a higher frequency of breastfeeding in an intervention group (*P* = 0.001) in which nurses assigned to an immunization service were trained to give 5 infant feeding messages in the central Mexican state of Morelos.

Low-burden breastfeeding programs are essential for sustainability, given the high workload of health personnel. When these interventions are integrated within existing primary care programs, they are more cost-effective [27]. Considering the real contexts of mothers’ interactions with health professionals, which tend to be limited in time, vaccination time can be selected to discuss infant feeding.

Other studies have used IC when vaccinating infants [24, 28]. In a study conducted in Australia [24], nurses assigned to the immunization service were trained to perform counseling following WHO guidance on breastfeeding. The sessions were held when the mothers had their children vaccinated at 2, 4 and 6 months of age. The counseling used a motivational interview tool, which was adapted to the mothers’ needs and decisions. Among the obstacles most frequently encountered were returning to work and community disapproval of breastfeeding in public. The results showed a significant increase in EBF at 4 months (*p* = 0.047) in the intervention group.

The postpartum period is a vulnerable time for women. If they are given too much information about all the possible obstacles to breastfeeding, by the time the discussion centers on what truly matters to them, their attention may have already been lost. Other studies have shown the effectiveness of doing counseling focused on women’s needs [23, 29, 30]. In a study implemented in the Women, Infants and Children (WIC) program in the USA [23], pre- and postnatal counseling was provided to pregnant women. The BAPT instrument was applied to assess their main problems and they were given personalized counseling based on that. The results showed a significant increase in EBF at 30 (*P* = 0.04) and 60 days postpartum (*P* = 0.002) in the intervention’s Hispanic subgroup. The authors conclude that it is an effective, adaptable, reproducible, and sustainable initiative to improve EBF rates in the WIC program.

It is striking that in our study, with just a short counseling session, there were significant breastfeeding changes. This is similar to a study conducted in Turkey [31], in which also only one counseling session increased EBF at 6 months (*p* = 0.04) and breastfeeding duration (*p* = 0.001). The major differences are that in the study from Turkey, the IC session was given at home, 3 days after delivery, and for a longer duration (30 mins). Despite the positive impact that a single IC session can have on EFB, a systematic review of breastfeeding counseling interventions [10] indicated that, the greatest effectiveness occurred with 4 or more sessions. Therefore, we plan to extend our pilot study to provide more interpersonal counseling sessions, each time mothers go to vaccinate their children at 2, 4, and 6 months.

Despite the efficacy shown in this study, we must be very cautious when interpreting the results since this was a pilot study in only one primary health care center, with a population of women who predominantly did not have paid employment. Due to the high mobility in Tijuana, only 71% of the sample could be followed at 2 months. There is a high risk of reporting bias given that we submitted the information for the trial registry retrospectively, and there was a delayed response due to the pandemic emergency. Other limitations include the lack of blinding in people who collected data at 2 months and a sample size that did not allow for subanalysis of effects by obstacle type.

The strengths of this study relied on following the Breastfeeding Gear Model recommendations of the evaluation conducted in Mexico [12]: 1) intervention based on formative research using a socioecological model 2) use of counseling tools that includes communication skills, 3) training of health personnel in primary care centers, 4) integration with other programs such as immunizations, and 5) evaluation of breastfeeding practices and intermediate variables of behavioral change (attitudes, self-efficacy).

## Conclusions

In this pilot study we found an improvement in EBF, attitudes, and self-efficacy 2 months after interpersonal counseling in a primary health care center. These results suggest that the promotion of breastfeeding utilizing a socioecological framework may improve breastfeeding rates by addressing the needs of women within their varying sociocultural contexts. The findings also highlight the importance of training health professionals with the knowledge and skills to clearly communicate breastfeeding messages. Future research should continue developing and evaluating programs that seek to increase breastfeeding rates in low- and middle-income countries.

### Future directions

The next phase for this project will include an expansion of this intervention to 10 health centers with the objective of evaluating the effectiveness of this program with a larger sample. Drawing on findings from the current study, the subsequent phase of this project will: 1) increase the sample size to conduct a subanalysis and evaluate the effect with specific obstacles, 2) add more variables for BF practices, to assess the effect of the intervention thoroughly (i.e., formula use, predominant BF, partial BF), 3) assess other variables from the TPB model (intentions, normative beliefs), 4) evaluate if mothers receive more information/support about breastfeeding in the 2 month follow-up period, and 5) increase the number of breastfeeding counseling sessions.

## Supplementary Information

**Additional File 1.** A word file with information about educational material used in the intervention (design and testing).

## Data Availability

The datasets during and/or analyzed during the current study available from the corresponding author on reasonable request.

## Abbreviations

EBF: Exclusive breastfeeding
BF: Breastfeeding
IC: Interpersonal counseling
PIM: Perception of insufficient milk
WHO: World health organization
BAPT: Breastfeeding attrition prediction tool

## References

[1] Lutter C. K, & Chaparro C. M. Neonatal Period: Linking best Nutrition Practices at Birth to Optimize Maternal and Infant Health and Survival. Food Nutr Bull. 2009; 30(2) _suppl2, S215–S224. 10.1177/15648265090302S205. 10.1177/15648265090302S205 20496614

[2] Kramer MS, Chalmers B, Hodnett ED, et al. Promotion of breastfeeding intervention trial (PROBIT): a randomized trial in the Republic of Belarus. JAMA. 2001; 285(4):413–420. 10.1001/jama.285.4.413. 10.1001/jama.285.4.413 11242425

[3] Horta B, Bahl R, Martines J, et al. Evidence on the long-term effects of breastfeeding: systematic reviews and meta-analyses. Study commissioned by WHO/CAH 2006.

[4] Bartick MC, Stuebe AM, Schwarz EB, Luongo C, Reinhold AG, Foster EM (2013). Cost analysis of maternal disease associated with suboptimal breastfeeding. Obstet Gynecol.

[5] Victora, Cesar G, et al. “Breastfeeding in the 21st Century: Epidemiology, Mechanisms, and Lifelong Effect.” Lancet. 2016; 387 (10017): 475–490. 10.1016/S0140-6736(15)01024-7. 10.1016/S0140-6736(15)01024-7 26869575

[6] Global breastfeeding scorecard, 2017. Tracking progress for breastfeeding policies and programmes. Global breastfeeding collective. UNICEF, WHO. https://www.who.int/nutrition/publications/infantfeeding/global-bf-scorecard-2017.pdf.

[7] González-Castell LD, Unar-Munguia M, Quezada-Sanchez A, et al. Situación de las prácticas de lactancia materna y alimentación complementaria en México: resultados de la ENSANUT 2018–19. Salud Publica Mex. 2020; 62: 704–713. https://www.saludpublica.mx/index.php/spm/article/view/11567. 10.21149/11567 33620967

[8] González de Cossío T, Escobar-Zaragoza L, González-Castell D, Reyes-Vázquez H, Rivera-Dommarco JA (2013). Breastfeeding in Mexico was stable, on average, but deteriorated among the poor, whereas complementary feeding improved: results from the 1999 to 2006 National Health and nutrition surveys. J Nutr.

[9] Olufunlayo TF, Roberts AA, MacArthur C, Thomas N, Odeyemi KA, Price M, et al. Improving exclusive breastfeeding in low and middle-income countries: a systematic review. Matern Child Nutr. 2019;15(3):e12788. 10.1111/mcn.12788. 10.1111/mcn.12788 PMC7199027 30665273

[10] McFadden A, Siebelt L, Marshall J.L, et al. Counselling interventions to enable women to initiate and continue breastfeeding: a systematic review and meta-analysis. Int Breastfeed J. 2019; 14 (42). 10.1186/s13006-019-0235-8. 10.1186/s13006-019-0235-8 PMC6805348 31649743

[11] Pérez-Escamilla R, Curry L, Minhas, et al. Scaling up of breastfeeding promotion programs in low- and middle-income countries: the "breastfeeding gear" model. Adv Nutr. 2012; 3(6): 790–800. 10.3945/an.112.002873. 10.3945/an.112.002873 PMC3648703 23153733

[12] Alonso A, Bonvecchio A, Colmenares M, et al. Becoming Breastfeeding Friendly. Reporte Índice País Amigo de la Lactancia Materna, Caso México 2016. Índice País Amigo de la Lactancia Materna: situación y recomendaciones para México. México: BBF; 2017. http://eventos.unkilodeayuda.org.mx/BBFMexico/docs/Reporte_BBFMexico.pdf. Accessed 5 June 2021.

[13] Bueno-Gutierrez Diana, Formative Research to Develop Breastfeeding Promotion Messages in Tijuana, Mexico. PhD Dissertation. University of California, Davis, 2014, 267; 3637802. https://pqdtopen.proquest.com/doc/1617974043.html?FMT=ABS.

[14] Ajzen I (1991). The theory of planned behavior. Organ Behav Hum Decis Process.

[15] Bueno-Gutierrez D, Armendariz-Anguiano AL, Romero-Castillo EU, Lopez-Vazquez K. Impact of breastfeeding messages on mothers’ attitudes. Breastfeeding medicine. 2017; suppl 1: S-9. https://www.liebertpub.com/doi/10.1089/bfm.2017.29058.abstracts.

[16] World Health Organization. Guideline: counselling of women to improve breastfeeding practices. Geneva: World Health Organization; 2018. Licence: CC BY-NC-SA 3.0 IGO. 30933442

[17] World Health Organization & United Nations Children's Fund (UNICEF). Estrategia Mundial para la Alimentación del Lactante y del Niño Pequeño. Genebra: Organización Mundial de la salud: 2003. https://apps.who.int/iris/handle/10665/42695. Accessed 6 June 2021.

[18] Janke J. Prediction of breastfeeding attrition: Instrument development. Appl Nurs Res. 1992; 5 (1): 48–53. 10.1016/S0897-1897(05)80086-2. 10.1016/s0897-1897(05)80086-2 1570961

[19] Dick M. J, Evans M. L, Arthurs J. B, et al. Predicting Early Breastfeeding Attrition. J Hum Lact. 2002; 18(1): 21–28. 10.1177/089033440201800104. 10.1177/089033440201800104 11845733

[20] Gill SL, Reifsnider E, Lucke JF, Mann AR (2007). Predicting breast-feeding attrition: adapting the breast-feeding attrition prediction tool. J Perinatal Neonatal Nursing.

[21] Swigart T, Bonvecchio A, Theodore F, et al. Breastfeeding practices, beliefs and social norms in low resource communities in Mexico: Insights for how to improve future promotion strategies. Plos One, 2017;12(7): e0180185. 10.1371/journal.pone.0180185. 10.1371/journal.pone.0180185 PMC5495390 28671954

[22] Morrow AL, Guerrero ML, Shults J, Calva JJ, Lutter C, Bravo J, et al. Efficacy of home-based peer counselling to promote exclusive breastfeeding: a randomized controlled trial. Lancet. 1999;353(9160):1226–31. 10.1016/S0140-6736(98)08037-4. 10.1016/S0140-6736(98)08037-4 10217083

[23] Edmunds LS, Lee FF, Eldridge JD, Sekhobo JP (2019). Outcome evaluation of the you can do it initiative to promote exclusive breastfeeding among women enrolled in the New York State WIC program by race/ethnicity. J Nutr Educ Behav.

[24] Elliott-Rudder M, Pilotto L, McIntyre E, Ramanathan S (2014). Motivational interviewing improves exclusive breastfeeding in an Australian randomised controlled trial. Acta Paediatr.

[25] Demirci JR, Bogen DL (2017). An ecological momentary assessment of Primiparous Women's breastfeeding behavior and problems from birth to 8 weeks. J Hum Lact.

[26] Monterrosa EC, Frongillo EA, González de Cossío T (2013). Scripted messages delivered by nurses and radio changed beliefs, attitudes, intentions, and behaviors regarding infant and young child feeding in Mexico. J Nutr.

[27] Bonuck KA, Lischewski J, Brittner M (2009). Clinical translational research hits the road: RCT of breastfeeding promotion interventions in routine prenatal care. Contemp Clin Trials.

[28] Patel A, Kuhite P, Puranik A. et al. Effectiveness of weekly cell phone counselling calls and daily text messages to improve breastfeeding indicators. BMC Pediatr. 2018; 18 (337). 10.1186/s12887-018-1308-3. 10.1186/s12887-018-1308-3 PMC6206669 30376823

[29] Zhu Y, Zhang Z, Ling Y, Wan H (2017). Impact of intervention on breastfeeding outcomes and determinants based on theory of planned behavior. Women Birth.

[30] Nilsson IMS, Strandberg-Larsen K, Knight CH, Hansen AV, Kronborg H (2017). Focused breastfeeding counselling improves short- and long-term success in an early-discharge setting: a cluster-randomized study. Matern Child Nutrition.

[31] Aksu H, Küçük M, Düzgün G (2011). The effect of postnatal breastfeeding education/support offered at home 3 days after delivery on breastfeeding duration and knowledge: a randomized trial. J Matern Fetal Neonatal Med.

