# Supplementary material for: Breastfeeding counseling based on formative research at primary healthcare Services in Mexico
Source: Int J Equity Health. 2021 Jul 27;20:173. doi: 10.1186/s12939-021-01491-6 (PMC8314515; doi:10.1186/s12939-021-01491-6)

**Appendix 1**

**Educational Material Design**

**Reference 13:** Bueno-Gutierrez Diana, Formative Research to Develop Breastfeeding Promotion Messages in Tijuana, Mexico. PhD Dissertation. University of California, Davis, 2014, 267; 3637802.

We used qualitative methods to design messages based on a socio-ecological framework and social marketing principles. Our message development model had 4 phases: 1) Breastfeeding obstacles assessment: We used a variety of qualitative methods to assess breastfeeding obstacles; 2) Message content development: Messages were designed by a panel of public health professionals and lay women; 3) Message refining and tailoring: Qualitative methods were used to identify message preferences for mothers; 4) Cognitive testing for final messages: We conducted cognitive response testing with mothers to evaluate how messages were processed and understood.


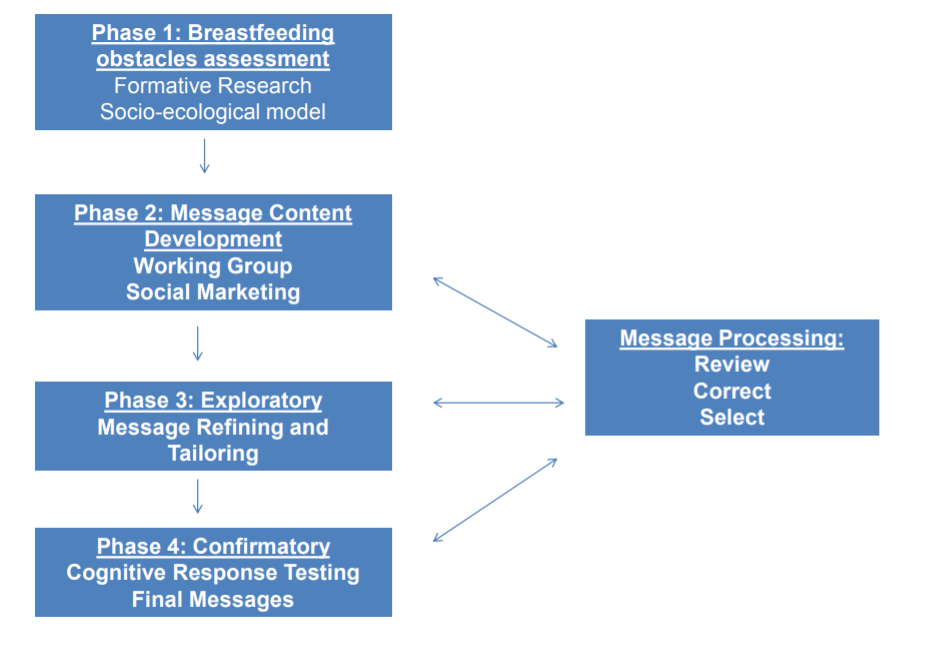


The message content and structure were developed by a panel that included nutrition and public health professionals, community leaders and lay women with experience supporting breastfeeding in their communities. After analyzing data from formative research that assessed the attitudes, beliefs, practices and obstacles to breastfeeding in these communities we designed 10 messages for women, taking into account the following characteristics: 1) Simplicity (easy to read); 2) Credibility, based on WHO recommendations combined with local formative research; 3) Practical/Actionable; 4) Motivational or emotionally-based; 5) Culturally sensitive; 6) Based on a socio-ecological framework, there were messages targeting individual, group and societal levels. These characteristics were chosen by the working team based on the literature review of successful social marketing campaigns and formative research conducted in the study communities.

**Message Refining**

One focus group and 23 interviews were conducted with 30 low-income mothers of children (≤5 years old). Sessions primarily focused on mothers' perceptions of messages and supporting content. We asked participants to select the message they liked the most in terms of how convincing, motivational and practical/ actionable they found it to be. All messages were presented in written form, read aloud, and discussed individually in a rotated order to prevent order bias. Participants discussed their reactions to the messages with the interviewer in the interviews and with other participants in the focus group. Additionally, participants reviewed the content and circled items they found interesting (ie, “liked”) and crossed out any information they did not like or did not understand. Participants discussed their preferences for content and potential areas of confusion.

**Cognitive Testing**

In a sample of 10 low-income mothers of children ≤5 years old, we used 3 approaches to elicit responses: 1) Think aloud, we asked participants to verbalize all of the thoughts they were thinking when examining the different messages; 2) Paraphrasing, participants were asked about the meaning of the message in their own words; and 3) Concurrent probing, we asked participants questions to further clarify their answers during the interview.

Final messages for mothers were similar to others from campaigns based on social marketing principles such as in Brazil they were: “you can produce enough milk’, “your breasts will not drop if you breastfeed”, “continue breastfeeding, every women can’’, and “make up your own mind” in recognition of the bias by many pediatricians for formula. The USDA Loving support campaign had “busy moms: breastfeeding works around my schedule”, “embarrassment: don’t shy away from breastfeeding”, “encouragement: give a breastfeeding mom your loving support”. The National Breastfeeding Awareness Campaign had “Babies were born to breastfeed” and Australia had “It’s OK to breastfeed….anywhere”.

In summary, we used the following strategies applied by successful breastfeeding campaigns based on social marketing:

- Using focus groups as a valuable method for understanding the perceptions, values, opinions, and attitudes of a potential target audience;
- Use of formative research that applies the social–ecological model to different population segments;
- Information from influential societal forces (family, health care providers, key informants from social areas) that affect women’s decision and ability to breastfeed;
- Engaging people on an emotional level and tailor self-efficacy messages in order to maximize the audience's confidence in their abilities (29, 65).

**Brochures and Posters for the Pilot Study**

There were 5 brochures for obstacles with more practical information

- Pain: How to get a good latch, when do you need to ask for professional support
- PIM: How do you know you are producing enough milk for your baby
- Infant dissatisfaction: Understand 3 normal infant behaviors: Crying and hunger-satiety cues, sleep, breastfeeding on demand (No schedule setting)
- Family support: How Dad/Grandparents can support a breastfeeding mother
- Work: Rights and regulations, milk expression

For the other 5 obstacles with a more psychological-social content we used posters with a main message and a picture with a “real breastfeeding mom” from Tijuana. These images were taken with permission from a local campaign and were pre-tested.

**Brochure about pain (Part 1)**


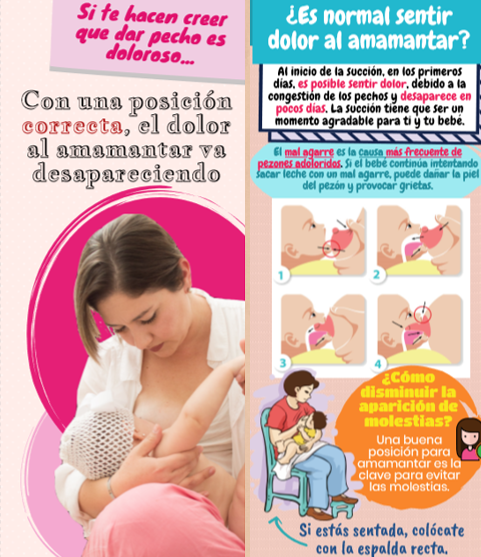


**Common section of all brochures**


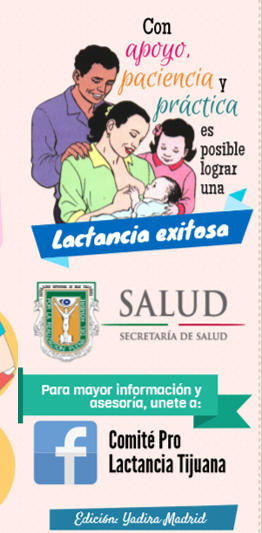


**Poster about breastfeeding in public**


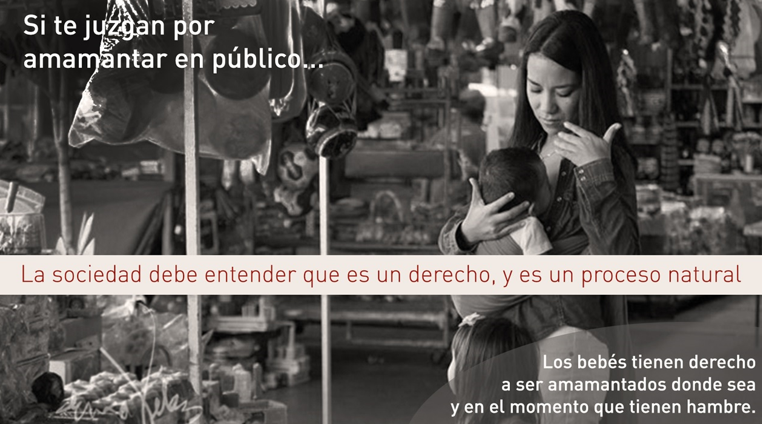

Supplement: Supplementary file 1 — Additional File 1. A word file with information about educational material used in the intervention (design and testing). [file 12939_2021_1491_MOESM1_ESM.docx]
